# 1019. Clinical Impact of a Rapid Cerebrospinal Fluid Diagnostic Stewardship Program for Suspected Central Nervous System Infections in Children

**DOI:** 10.1093/ofid/ofab466.1213

**Published:** 2021-12-04

**Authors:** Kevin Messacar, Claire Palmer, LiseAnne Gregoire, Audrey Elliott, Elizabeth Ackley, Ken Tyler, Samuel R Dominguez, Samuel R Dominguez

**Affiliations:** 1 University of Colorado/ Children’s Hospital Colorado, Denver, Colorado; 2 Children’s Hospital Colorado, Aurora, Colorado; 3 George Washington University, Denver, Colorado; 4 University of Colorado, Aurora, Colorado; 5 University of Colorado, School of Medicine, Aurora, CO

## Abstract

**Background:**

Despite widespread use, the optimal implementation and clinical impact of FilmArray Meningitis Encephalitis Panel (MEP; Table 1) multiplex PCR testing of cerebrospinal fluid (CSF) in children with suspected (CNS) infections is unknown.

Table 1: FilmArray Meningitis Encephalitis Panel Test Characteristics

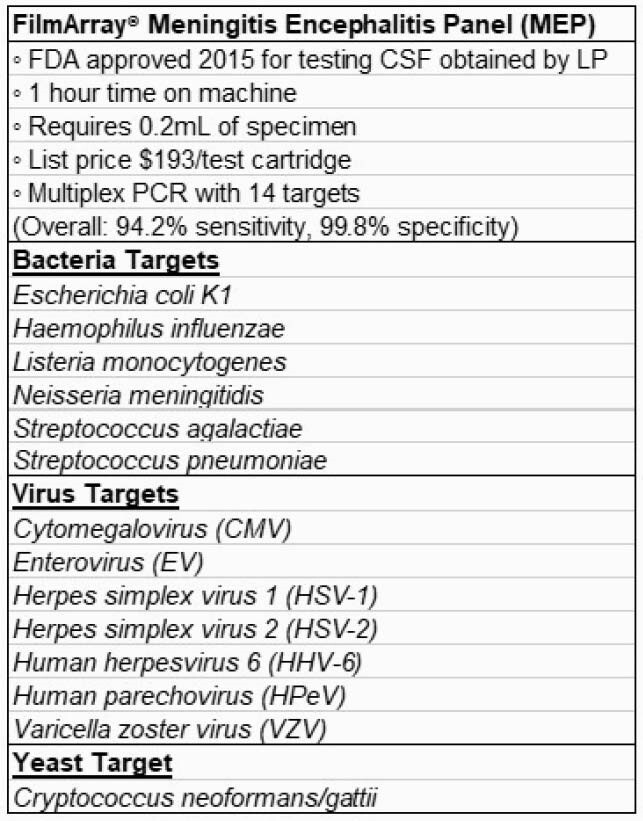

**Methods:**

A pre-post quasi-experimental cohort study to investigate the impact of implementing MEP using a rapid CSF diagnostic stewardship program was conducted at Children’s Hospital Colorado (CHCO). MEP was implemented with EMR indication selection to guide testing to children meeting approved use criteria: i. infants < 2mo, ii. immunocompromised, iii. encephalitis, iv. > 5 WBCs in CSF. Positive results were communicated with antimicrobial stewardship real-time decision support (Fig 1). All cases with CSF obtained by lumbar puncture (LP) sent to the CHCO microbiology laboratory meeting any of the 4 criteria above were included with pre-implementation controls (2015-2016) compared to post-implementation cases (2017-2018). Primary outcome was time-to-optimal antimicrobials (time from LP to 1^st^ dose of antimicrobials targeted to identified pathogen, or cessation when no treatable pathogen identified).

Figure 1: Rapid Cerebrospinal Fluid Diagnostic Stewardship Program Intervention Design

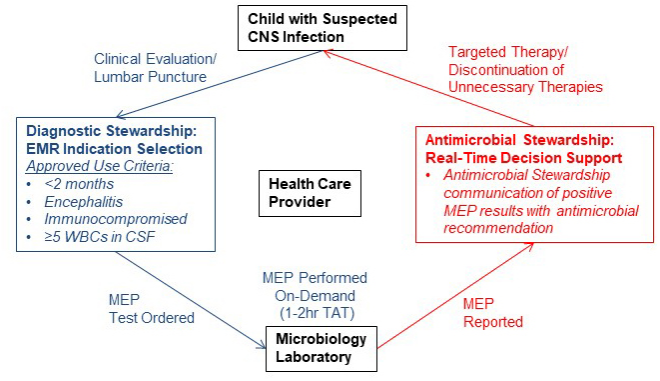

**Results:**

Post-implementation (n=1127) and pre-implementation (n=1124) group characteristics are in Table 2. Following implementation, MEP was sent in 72% of cases, largely replacing pathogen-specific singleplex CSF testing (Table 3). Time-to-optimal antimicrobials decreased by 10 hours (p< 0.0001; Fig 2). There were no differences in time-to-effective antimicrobials, hospital admissions, antimicrobial starts or length of stay. Time-to-positive CSF results was faster (4.8 vs. 9.6 hrs, p< 0.0001), IV antimicrobial duration was shorter (24 vs 36 hrs, p=0.004) with infectious neurologic diagnoses more frequently identified (15% vs. 10%, p=0.03). Overall, 3% had bacterial and 9% viral CNS infection identified. Enterovirus (n=128) was most common, then HSV (n=28) and parechovirus (n=17) with similar detection rates between groups

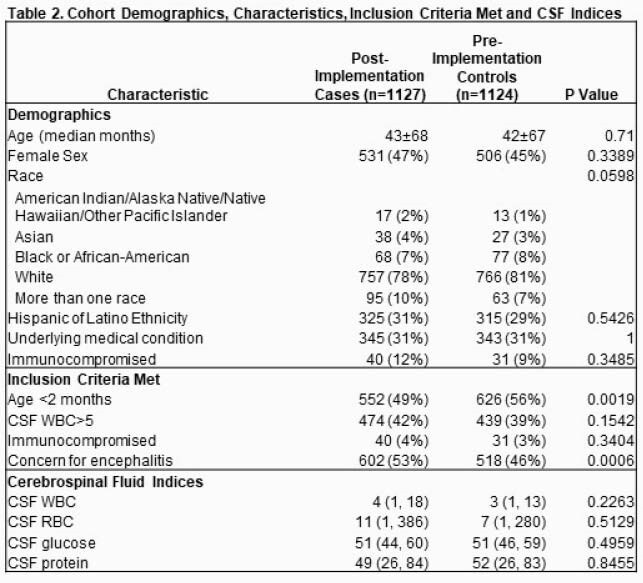

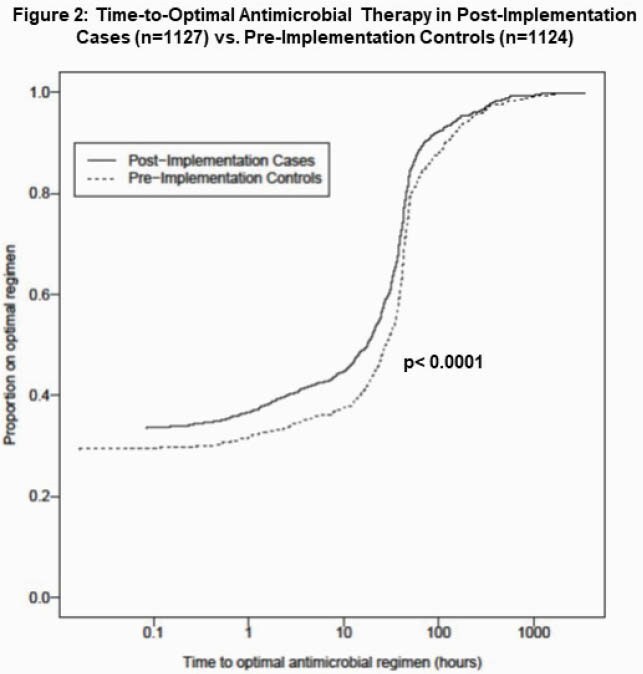

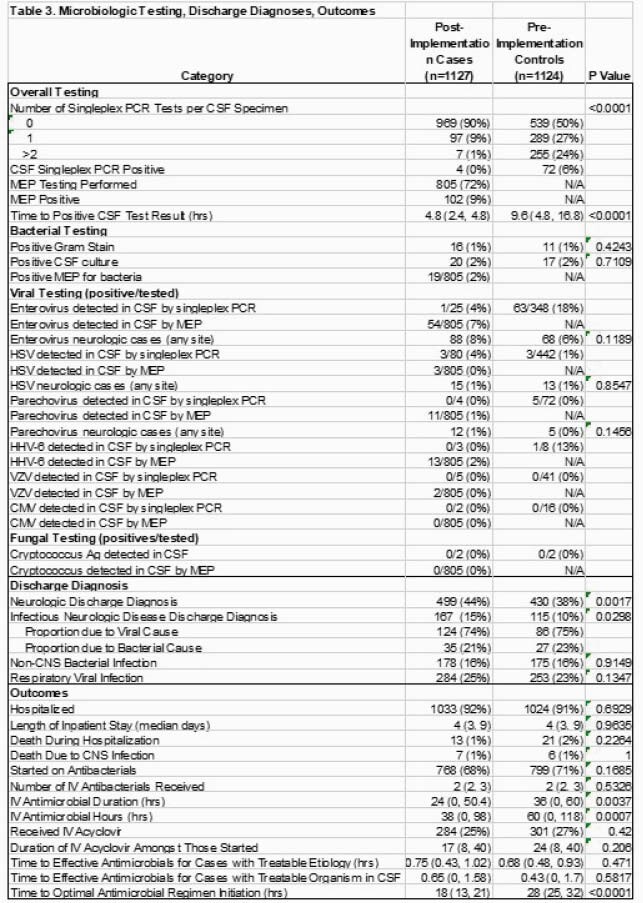

**Conclusion:**

Implementation of MEP with a rapid CNS diagnostic stewardship program improved antimicrobial use with faster results shortening empiric therapy. Routine MEP testing in high-yield cases rapidly detects common viral causes and rules out bacterial targets to enable antimicrobial optimization

**Disclosures:**

**Samuel R. Dominguez, MD, PhD**, **BioFire Diagnostics** (Consultant, Research Grant or Support)**DiaSorin Molecular** (Consultant)**Pfizer** (Grant/Research Support) **Samuel R. Dominguez, MD, PhD**, BioFire (Individual(s) Involved: Self): Consultant, Research Grant or Support; DiaSorin Molecular (Individual(s) Involved: Self): Consultant; Pfizer (Individual(s) Involved: Self): Grant/Research Support

